# Learning U-Net Based Multi-Scale Features in Encoding-Decoding for MR Image Brain Tissue Segmentation

**DOI:** 10.3390/s21093232

**Published:** 2021-05-07

**Authors:** Jiao-Song Long, Guang-Zhi Ma, En-Min Song, Ren-Chao Jin

**Affiliations:** School of Computer Science and Technology, Huazhong University of Science and Technology, Wuhan 430074, China; jiaosonglong92@163.com (J.-S.L.); esong@hust.edu.cn (E.-M.S.); jrc@hust.edu.cn (R.-C.J.)

**Keywords:** magnetic resonance images, brain tissue segmentation, multi-scale feature learning, multi-branch pooling, multi-branch dense prediction, multi-branch output

## Abstract

Accurate brain tissue segmentation of MRI is vital to diagnosis aiding, treatment planning, and neurologic condition monitoring. As an excellent convolutional neural network (CNN), U-Net is widely used in MR image segmentation as it usually generates high-precision features. However, the performance of U-Net is considerably restricted due to the variable shapes of the segmented targets in MRI and the information loss of down-sampling and up-sampling operations. Therefore, we propose a novel network by introducing spatial and channel dimensions-based multi-scale feature information extractors into its encoding-decoding framework, which is helpful in extracting rich multi-scale features while highlighting the details of higher-level features in the encoding part, and recovering the corresponding localization to a higher resolution layer in the decoding part. Concretely, we propose two information extractors, multi-branch pooling, called MP, in the encoding part, and multi-branch dense prediction, called MDP, in the decoding part, to extract multi-scale features. Additionally, we designed a new multi-branch output structure with MDP in the decoding part to form more accurate edge-preserving predicting maps by integrating the dense adjacent prediction features at different scales. Finally, the proposed method is tested on datasets MRbrainS13, IBSR18, and ISeg2017. We find that the proposed network performs higher accuracy in segmenting MRI brain tissues and it is better than the leading method of 2018 at the segmentation of GM and CSF. Therefore, it can be a useful tool for diagnostic applications, such as brain MRI segmentation and diagnosing.

## 1. Introduction

The segmentation of brain tissues from magnetic resonance (MR) images is of primary importance for subsequent diagnosis, pathological analysis, prognosis assessment, and brain development monitoring [1]. MR images have different kinds of modalities, including T1, T1C, T2, PD, T1IR, and FLAIR, and each reflects particular characteristics of tissue regions in brain.

For example, both T2 and FLAIR sequences describe low signals in the white matter region and high signals in the gray matter region. T2 depicts marked high signals for the cerebrospinal fluid, where FLAIR shows low or no intensity signals [2,3]. Hence, we can aggregate these multiple modalities to capture richer information to improve brain tissue segmentation performance.

Generally, the goal of brain segmentation is to classify brain voxels as three major brain structures: gray matter (GM), white matter (WM), and cerebrospinal fluid (CSF). Traditional manual segmentation is time-consuming and tedious, and it is easy to produce bias due to the operator’s subjective experience. Thus, the research on automatic brain tissue segmentation algorithm has been receiving extensive attention [4,5,6,7].

A few machine learning methods for automatic brain tissue segmentation have been proposed in literature, including methods based on hand-crafted features [7,8,9,10] and methods based on multi-atlas registration [11,12]. However, the performances of these methods are limited, owing to the fuzzy brain tissue edge [13], the multi-source noise, and the inhomogeneous intensity in brain MR images.

Recently, deep learning has been extensively applied in medical image segmentation; for example, segmenting local lesions such as tumors [14,15,16] and organs such as brain tissues [5,17,18]. By pooling features with different resolutions in the encoding path and recovering sharp object boundaries in the decoding path, the U-Net [19] can capture rich contextual information because of this encoding-decoding manner. The U-Net framework and its extensions have become the most common deep neural networks used in medical image segmentation.

**Figure 1:**
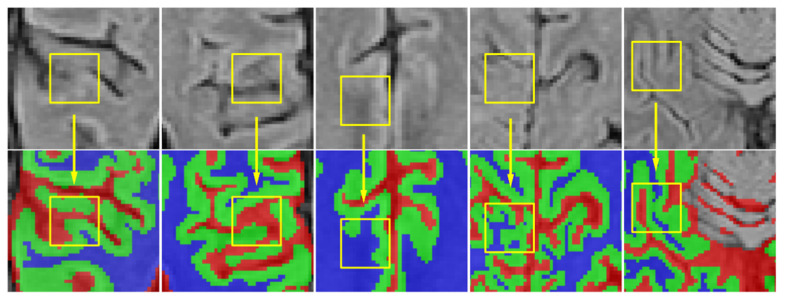
Illustration of complex anatomical structures and variable shapes in MRBrain2013S dataset. The first row lists the brain MR images in different areas. The second row shows the corresponding ground truth labels, where the colors denote different regions of the brain: red represents the cerebrospinal fluid (CSF), green the gray matter (GM), and blue the white matter (WM). Other tissues are represented with gray.

However, it still faces challenges considering the complex anatomical structures and variable shapes of brain tissues. Five examples are shown in Figure 1, where the intensity of white matter is similar to the gray matter in the rugged edge (in the yellow box), hence, it is difficult to segment these brain tissues successfully because of the description of confused boundaries.

In terms of segmenting brain tissues accurately, we discovered that the problem of the U-Net-based models is the lack of multi-scale context information with a suitable receptive field. Unfortunately, the exploitation of multi-scale CNN features for semantic segmentation is a challenging task.

Conventionally, the multi-scale technique can be divided into two typical strategies: pooling at multiple scales and convoluting at multiple fields-of-views. For the former, [20] applies pooling operations with different grid scales. However, without a suitable number of grid scales, the detailed boundary information will be lost. For the latter, mainstream methods [21,22] adopt multiple rates of atrous convolution with a larger receptive field to harness multi-scale context information. However, although they can capture global information by multiple rates of atrous convolution, it is easy to encourage irrelevant redundant information [23] if without a suitable receptive field. In [21,22], extracting the multi-scale information is encoded in the last feature map; however, extracting multi-scale information in the previous feature layer is equally important, especially in medical image processing.

In addition, the above methods focus on extracting the multi-scale feature information on the spatial dimension. To learn better feature representation, the channel dimension-based multi-scale feature extracting is crucial; however, the related study is still lacking. Zhang et al. [24] suggest that a structure called “Densely Adjacent Prediction” might be used to encode spatial information into channels, and utilizes the adjacent channel information to predict results; however, it lacks the complementary multi-scale features [25].

To solve the aforementioned problems, https://orcid.org/0000-0001-7365-0053, (accessed on 29 April 2021) jointly obtain high-precision multi-scale CNN features. In this work, we propose to segment brain tissues with a novel Multi-scale Spatial and Channel Dimension U-Net (MSCD-UNet).

Our proposed architecture is based on UNet and influenced by the information extractors named multi-branch pooling (MP) and multi-branch dense prediction (MDP). To overcome the limitation of the 3D-UNet network, we propose a novel network by embedding the MP and MDP into 3D-UNet. The embedded network can capture more context cues while enhancing the details of multi-scale information by using the extractor MP in the encoding part and recovering the corresponding localization to a higher resolution layer by using the extractor MDP in the decoding part. Extensive experiments on three benchmarks with MRBrain2013, IBSR18, and ISeg2017 datasets demonstrate that our approach performs competitively against other state-of-the-art methods. The contributions of our paper are itemized in the following:

1. We have proposed a novel network by introducing spatial dimension and channel dimension-based multi-scale CNN feature information extractors into its encoding-decoding framework. In the encoding part, we propose the multi-branch pooling information extractor, called MP, to capture multi-scale spatial information for the information compensating. As pooling is easy to lose the useful spatial information when the feature map resolution is reduced, we propose the MP by using multiple max pooling with different kernel sizes in parallel to reduce the information missing and collect the neighborhood information with a suitable receptive field;
2. In the decoding part, we propose the multi-branch dense prediction, an information extractor, called MDP, to capture multi-scale channel information for the information compensating. During the decoding phase, after the maps resolution upsizing, the spatial information in these decompressed feature maps is fixed and the detailed information is represented more in channel dimension, so we consider that the prediction results at the adjacent position are related to the result of the center position. We divided the prediction result into multiple channel groups, and the multi-scale channel information of the center position can be created by averaging these groups for the purpose of information compensation. In addition, we designed a multi-branch output structure with MDP in the decoding part to form more accurate edge-preserving predicting maps by integrating the dense adjacent prediction features at different scales.

The two proposed ideas are first used in this paper. We carry out extensive experiments on three benchmarks (MRBrainS12, IBSR18, and ISeg2017) to evaluate our method. The results have proved the feasibility of our proposed method and the performance of improvement.

The remainder of the paper is structured as follows. The related work of brain tissue segmentation is described in Section 2. In Section 3, a detailed scheme of our solution is presented, including spatial-based multi-scale feature extractor in encoding, channel-based multi-scale feature extractor in decoding, multi-branch output structures, and MSCD-UNet. We perform MSCD-UNet experiments with MRBrain2013, IBSR18 and ISeg2017 datasets in Section 4, and discuss the results in Section 5. Finally, we conclude the paper with future work suggestions in Section 6.

## 2. Related Works

In this section, we briefly describe the related work of MRI brain tissue segmentation. Subsequently, we list the typical brain segmentation approaches in three categories: atlas-based registration, traditional machine learning-based, and deep learning-based. Atlas-based approaches are widely used in multi-modal circumstances [26,27]. These methods rely on registering several atlases to the target image, and then propagating the manual labels to this image. The label fusion strategy [28,29,30] is used to adjust the registered labels of different atlases to form the final segmentation. Because the accuracy of the registration processing is the key affecting the final segmentation result, it needs a large number of target templates to adapt the difference of brain anatomy, and these approaches are computationally expensive and perform poorly.

To address the above problems, many traditional methods based on machine learning are applied to segment brain tissues. For example, [31] adopted both intensity and spatial features to complete brain segmentation by using support vector machine. Tong et al. [17] used discriminative dictionary learning and sparse coding techniques to label brain tissues. Wang et al. [32] effectively integrated 3D Haar-like features from multi-source images together by utilizing the random forest technique to perform tissue segmentation. Zhang et al. [33] proposed a novel hidden Markov random field (HMRF) model which can encode spatial information through the mutual influences of neighboring sites to improve its accuracy and robustness. K. Mishro et al. [34] proposed a type-2 AWSFCM clustering algorithm to perform segmentation tasks. It assigned the problematic equidistant pixels to a single cluster by offering larger weight to pixel closing to the expected decision boundary. However, the main limitation of these traditional methods is that the intensity profiles of more detailed brain tissues overlap [16], and it is hard to distinguish between tissues in different brain regions.

Recently, deep learning methods based on CNN have become a powerful tool for segmenting brain tissues, which can overcome the drawback of atlas-based registration and traditional machine learning models. Zhang et al. [35] trained a CNN model for infant brain tissue segmentation by harnessing 2D single patches on axial plane slices of T1, T2, and FLAIR images. Moeskops et al. [36] introduced multiple patch sizes and multiple convolution kernel sizes into CNN to obtain multi-scale information to recognize the detailed information for brain tissue segmentation. Chung et al. [37] proposed to combine the dynamic random walker with the decay region of interest into CNN to acquire smooth segmentation of subcortical structures. However, these patch-based voxel classification methods still face troubles such as the limitation of local information and the complexity of boundaries surrounded by adjacent voxels.

Recently, fully CNN (FCNN) has been widely applied in brain segmentation to solve the above problems, as they predict the labels of voxels within the input patch simultaneously. Nie et al. [38] trained a shared network for each modality image, then fused their high-layer features in the final predicting layer. Xu et al. [39] regarded three serial slices as input of three channels to predict the middle slice by using the fully CNN. Chen et al. [40] proposed a model named VoxResNet to segment brain MR images, which can jointly encourage features of high-level context information and low-level image appearance to compensate the missing information at different levels. Dolz et al. [41] proposed HyperDenseNet, which can learn more complex combinations between modalities to expand the learning ability of all levels of abstraction and representation. Li et al. [42] captured and aggregated multi-scale features of brain tissues by using a multi-modality aggregation network named MMAN to accomplish brain segmentation with better accuracy. Chen et al. [43] presented a Dense-Res-Inception network to segment the cerebrospinal fluid, which is able to produce distinct features in terms of intensity, location, shape, and size. Lei et al. [44] proposed a dual aggregation network to adaptively aggregate different information of infant brain MRI modalities. Qamar et al. [18] proposed to combine dense connection, residual connection, and inception module to achieve excellent results. Yu et al. [45] developed a densely connected 3D-DenseVoxNet to preserve maximum information flow to ease the network training. Taoc et al. [46] presented a network very deep in architecture based on dense convolution network for volumetric brain segmentation. They used a model of bottleneck with compression to reduce the number of feature maps in each dense block, so as to reduce the number of learned parameters and result in computational efficiency. Dolz et al. [47] proposed a FCNN that adopts 3D spatial context of triplanar data and both global and local information for MRI brain segmentation. Sun et al. [48] proposed a volumetric feature recalibration (VFR) layer, which could richly capture the spatial contextual information, then leverage it for volumetric weighting between spatial layers.

An in-depth summarization of some of the related works in brain MRI segmentation along with techniques, advantages, and limitations is documented in Table 1.

In this paper, we present a 3D U-Net-based architecture that includes multi-branch pooling and multi-branch dense prediction to capture the multi-scale features, which are the important factors that enable a FCNN to capture the complex contextual information and enlarge its limited receptive field.

## 3. Materials and Methods

**Figure 2:**
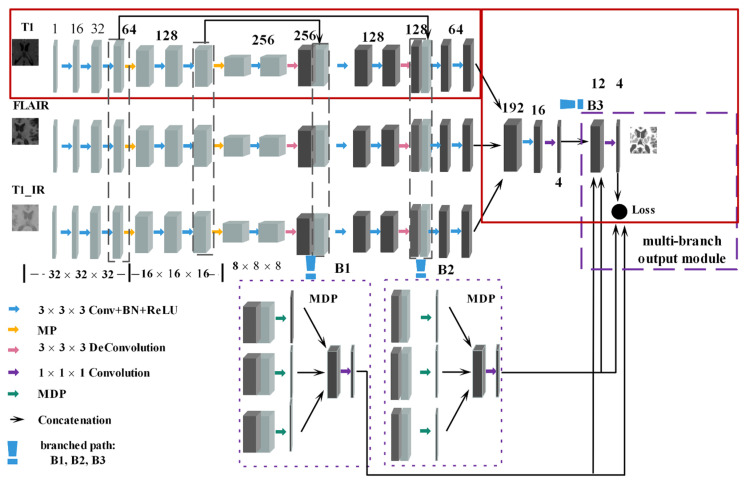
The architecture of proposed MSCD-UNet for brain segmentation consisting of MP, MDP, and the multi-branch output module. Three input samples with size 32 × 32 × 32 were randomly cropped with a same center point from 3 modalities (T1, T2-FLARI, T1_IR), they have the corresponding position information. Solid red box represents the subnetwork for T1 MR image.

Deep learning, one of the most effective methods in computer vision, is widely used. As illustrated in Figure 2, we designed a novel, fully convolutional neural network (FCNN) constructed by a 3D UNet with the proposed feature information extractors (MP and MDP). The proposed network is called Multi-scale MSCD-UNet. The details of the proposed approach are listed in the next subsection.

### 3.1. Model Overview

In Figure 2, the input slices were randomly cropped with the same center point from 3 modalities (T1, FLARI, T1_IR); thus, they have the corresponding position information. The concrete architecture of the MSCD-UNet consists of three main modules: MP, MDP, and multi-branch output. We exploit MSCD-UNet to capture the rich multi-scale semantic information in the encoding path by using multiple max pooling with different kernel sizes in parallel, and allow the detailed object boundary recovering in the decoding path by dividing the dense prediction maps into multiple groups. For each scale in the decoding path, we use a concatenation operation to connect these dense prediction maps for the information compensating. The multi-branch output module under a deeply supervised network component aims at largely discovering the learning ability of CNN from bottom to top layers, and producing more precise segmentation results by integrating the predicting maps of identical size at the last layer.

### 3.2. Multi-Branch Pooling and Multi-Branch Dense Prediction

The information loss of down-sampling and up-sampling operations of an FCNN-based model is a common problem, which is mentioned as the weak ability of feature extracting in the encoding and decoding paths. In the encoding path, the repeated accumulation of pooling and convolution with strides at consecutive layers meaningfully reduces the spatial resolution of feature maps, then causing a loss of spatial information. In the decoding path, deconvolutional layers have been used to recover the corresponding localization for the higher resolution layer; it will result in great losses in channel dimension. In order to enhance the ability of feature extracting in spatial and channel dimensions, we propose to utilize a multi-scale spatial and channel dimensions-based network to capture higher semantic information during encoding and gradually recover the spatial information during decoding.

**Figure 3:**
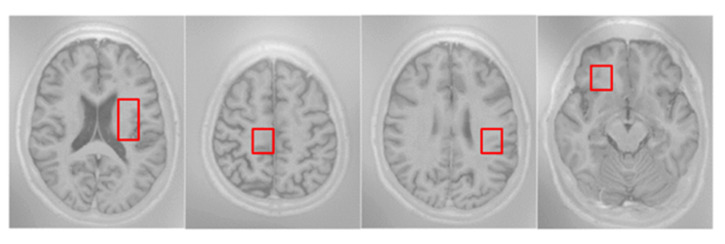
Example of the modality of T1_IR from patient no. 5 MRI. In this example, the intensities of different brain tissue in the different local brain regions are close to each other, like the examples in the red boxes.

**Figure 4:**
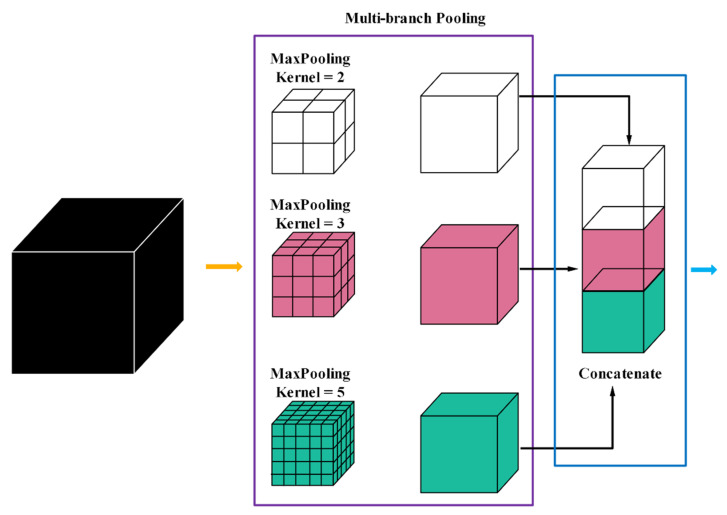
Encoding path with multi-branch max pooling.

Multi-branch pooling (MP): pooling is employed to improve the invariants of the transformed image, the compact representations of semantic information, and the better robustness to noise and clutter [49]. The size of the feature map can be reduced by using different pooling scales, which will effectively ensure the validity of information and speed up the calculation. Empirically, max-pooling is widely used in the field of medical image processing; however, it is easy to lose the useful spatial contextual information when the feature map resolution is reduced. In order to reduce the loss of information, inspired by [20], they have adopted multiple rates of atrous convolution in parallel to harness multi-scale context information. However, although they can capture global information by multiple rates of atrous convolution, it is easy to encourage irrelevant redundant information without a suitable receptive field. Thus, we propose multi-branch pooling to collect the multi-scale spatial information during the encoding procedure, which in parallel consists of multiple max pooling with different kernel sizes. The parallel max-pooling separates the feature maps into different adjacent regions and produces pooled representations for the same location, while the neighborhood information with a suitable receptive field can be captured for the information compensating. After the MP operation, these parallel feature maps pooled with different kernels finally have identical size, and each time the feature map size is reduced by factor of two. In addition, we can see from Figure 3, the intensities of different brain tissues in different local regions of the brain are close to each other; thus, a lot of redundant information will be produced by using atrous convolution with a large receptive field. However, the proposed MP, as illustrated in Figure 4, can capture the multi-scale context information with a suitable receptive field.

Our proposed MP contains a three-branch structure with bin size 2 × 2 × 2, 3 × 3 × 3, and 5 × 5 × 5 in first pooling stage, and a two-branch structure with bin size 2 × 2 × 2 and 3 × 3 × 3 in last pooling stage. The key idea of MP is to use suitable kernels, whose size is controlled by the parameter K. In order to gain the optimal combination of kernel size K, we enumerate different kernel sizes and validate the performance respectively; the results are detailed in Section 4.1. Additionally, we perform extensive experiments to compare the performance between the max pooling and the average pooling in Section 4.1.

**Figure 5:**
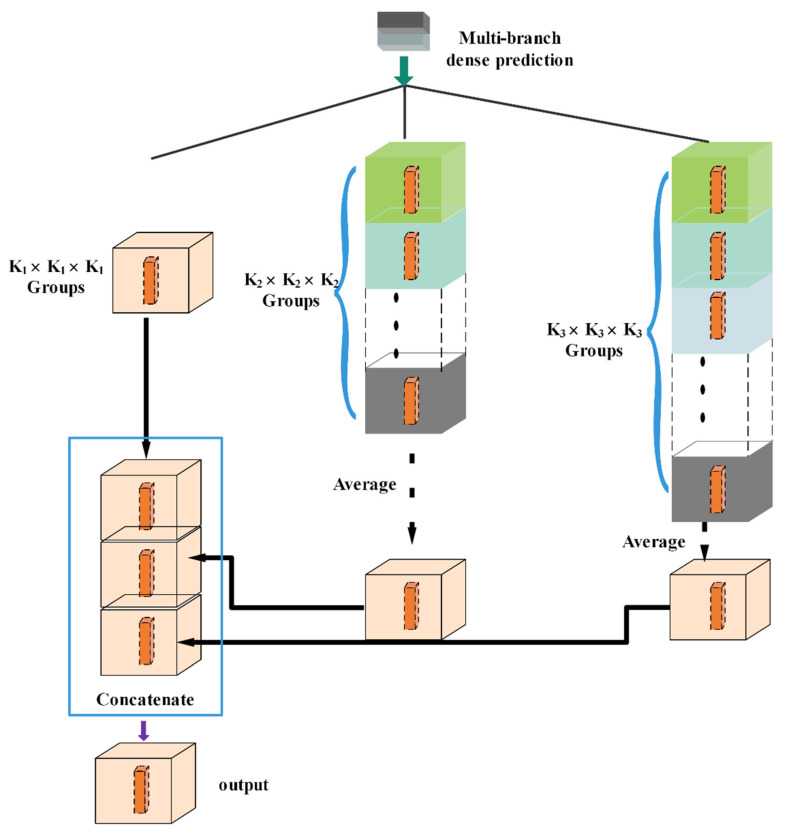
The components of multi-branch dense prediction (MDP).

Multi-branch dense prediction (MDP): as in the work of [19], the decoding module consists of a series of simple bilinear up-samplings by a consecutive factor of 2, which could be regarded as a naive decoding module. However, this naive decoding module may not fully recover the segmented object details. During the decoding phase, the compressed feature maps from the deepest encoding layer will be used to recover feature maps resolution by using deconvolution and up-sampling operation. After the maps resolution upsizing, the spatial information in these decompressed feature maps is fixed so the detailed information is represented more in channel dimension; thus, it implies we will be supposed to focus on the collection of complex information in channel dimension. Inspired by [24], considering that the predict results at the adjacent position are related to the result of the center point, they have divided the feature channels into one group in each up-sampling operation, where the number of feature channels has been fixed, resulting in a loss of information. In order to enhance the ability of feature extracting in channel, we design a channel-based multi-scale feature extractor (see Figure 5), named MDP, in which the feature channels are divided into multiple groups to free the fixed feature channels; the result of center point can be created by averaging these groups for the information compensating.

For the decoding path, the feature point at the spatial location $\left(l,n,m\right)$ is responsible for its semantic information. In order to collect as much spatial information as possible into channels, this information extractor can be considered to predict results at the adjacent position, e.g., $\left(l-1,n+1,m+1\right)$. When obtaining the final predicted results, results at the center position $\left(l,n,m\right)$ can be created by averaging the related scores. Concretely, supposing that the three window sizes are$\;k_{1}\times k_{1}\times k_{1},\;k_{2}\times k_{2}\times k_{2},\;k_{3}\times k_{3}\times k_{3}$, respectively, we divided the feature channels into three groups $k_{1}\times k_{1}\times k_{1},\;k_{2}\times k_{2}\times k_{2},\;k_{3}\times k_{3}\times k_{3}$, respectively. The outputs of MDP R are formed as follows:

(1) $$R_{l,m,n}^{k1}=\frac{1}{k_{1}\times k_{1}\times k_{1}}\;\underset{0\leq r,s,t<k_{1}}{\sum }y_{l+r-\lfloor \frac{k_{1}}{2}\rfloor ,\;\;m+s-\lfloor \frac{k_{1}}{2}\rfloor ,\;\;n+t-\lfloor \frac{k_{1}}{2}\rfloor }^{\left(r\times k_{1}+s+t\right)},$$

(2) $$R_{l,m,n}^{k2}=\frac{1}{k_{2}\times k_{2}\times k_{2}}\;\underset{0\leq r,s,t<k_{2}}{\sum }y_{l+r-\lfloor \frac{k_{2}}{2}\rfloor ,\;\;m+s-\lfloor \frac{k_{2}}{2}\rfloor ,\;\;n+t-\lfloor \frac{k_{2}}{2}\rfloor \;}^{\left(r\times k_{2}+s+t\right)},$$

(3) $$R_{l,m,n}^{k3}=\frac{1}{k_{3}\times k_{3}\times k_{3}}\;\underset{0\leq r,s,t<k_{3}}{\sum }y_{l+r-\lfloor \frac{k_{3}}{2}\rfloor ,\;\;m+s-\lfloor \frac{k_{3}}{2}\rfloor ,\;\;n+t-\lfloor \frac{k_{3}}{2}\rfloor }^{\left(r\times k_{3}+s+t\right)},$$

where $R_{l,n,m}$ represents the result at the position$\;\left(l,n,m\right)$ and $y_{l,n,m}^{c}$ is the feature map at position $\left(l,n,m\right)$ belonging to channel group c. The MDP scheme is illustrated in Figure 5.

We employed MDP as the output of our decoding module (see Figure 2). We set $k_{1}=1,\;k_{2}=3,\;k_{3}=4$ to conduct our experiments. In order to prove the validity of MDP, we tested the baseline model U-Net only with $k_{1}=1$ in the experimental section, and the results show that the MDP can improve the final performance. The results are detailed in Section 4.2.

### 3.3. Multi-Branch Output Modules and Loss Functions

The idea of multi-branch output modules is widely used in the deeply supervised network. In view of our proposed network, collecting multi-scale information in the decoding path can encourage more reliable and accurate predictions of the final results. Thus, we integrate multiple branch output in each scale after MDP operation (see Figure 2 for an illustration). Concretely, given a total H branch output, each output will generate the prediction by an up-sampling operation with the associated weights. The multiple loss function of the whole network can be defined as a weighted sum of all of the branch output loss; its calculation formula is as follows:

(4) $$Loss_{side}\left(W,w,gT\right)=\overset{H}{\underset{h=1}{\sum }}\beta_{h}l_{side}^{h}\left(W,w^{h},gT\right),$$

where $\beta_{h}$ stands for the weight of the $h_{th}$ output loss function, $l_{side}^{h}$ is the cross-entropy loss function, and the count of the additional output H is set to 3. $l_{side}$ is unfolded with the following formula:

(5) $$l_{side}\left(W,w^{h},gT\right)=-\underset{i\in gT}{\sum }\underset{c}{\sum }\omega_{c}gT_{c}logP\left(W,\;w^{h}\right),$$

where gT is the label of ground truth, c denotes the $c_{th}$ classification label and $\omega_{c}$ is the associated weight, and $P\left(\cdot \right)$ indicates the output of network as the probabilistic prediction in the $c_{th}$ output way. Finally, a fusion layer can be applied to aggregate the prediction from each additional output by:

(6) $$Loss_{fuse}\left(W,w,f\right)=\emptyset \left(gT,\sigma \left(\overset{H}{\underset{h=1}{\sum }}f_{n}Ap_{side}^{h}\right)\;\right),$$

where $f_{n}$ represents the fusion weight, $Ap_{side}^{h}$ indicates the activation of the $h_{th}$ output way, σ denotes the softmax activation function, and ∅ is the cross-entropy loss function. Finally, the final loss function of the network can be formed as:

(7) $$Loss_{final}=Loss_{fuse}\left(W,w,f\right)+Loss_{side}\left(W,w\right).$$

### 3.4. Network Architecture

The U-Net [19] has been widely applied in medical image segmentation, which adequately combines the low-level high resolution and the high-level low resolution feature maps. Our proposed MSCD-UNet is similar to the 3D-UNet [50], but it can make up for the deficiency of information missed in U-Net by using MP and MDP to capture rich multi-scale context information.

The architecture of MSCD-UNet in this paper is shown in Figure 2. We follow the strategy in [48], where sub-volumes of 32×32×32 are used as input for training. Instead of using the standard 3D U-Net with multi-channel inputs, we use a parallel feed forward network with different modalities and fuse their deep-high level features for voxel-wise prediction. The parallel feed forward network consists of three parts: input part, encoding part, and decoding part. The input part is divided into three parallel paths where the input data are T1, T2-FLAIR, and T1-IR, respectively. The encoding part includes two stages, each stage contains two 3×3×3 convolution layers and each is followed by a batch normalization (BN) and a non-linear activation function (ReLU). At the end of each stage, the MP is attached to reduce resolution. The number of feature channels is doubled after each stage. Similarly, the decoding part also contains two stages, each stage consists of a deconvolution layer of 2×2×2 followed by BN and ReLU. There are also two 3×3×3 convolution layers each followed by BN and ReLU. Additionally, MDP is used to collect complex multi-scale channel information to recover the corresponding localization to higher resolution layer in each stage. Finally, a fusion layer can integrate the prediction result from each MDP output to produce more accurate edge-preserving segmentation results.

### 3.5. Dataset Introduction

Our proposed method is successful on the MRBrainS13 dataset of brain segmentation challenge. The method is evaluated in this section by three different datasets: MRBrainS13, IBSR18, and ISeg2017.

**Figure 6:**
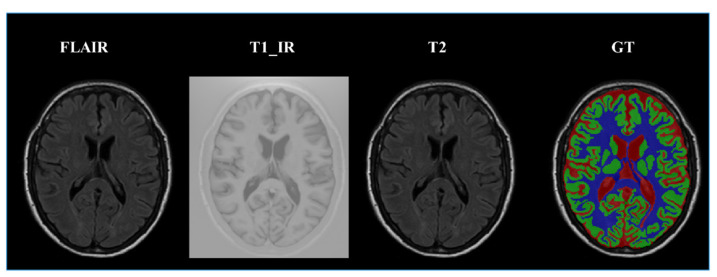
Example of MR images with different image modalities and the labels manually marked by experts; the first three images from left to right are FLAIR, T1, and T1_IR. The fourth image is the ground truth labels where the colors denote different regions of brain tissues: red represents cerebrospinal fluid (CSF), green the gray matter (GM), and blue the white matter (WM). Gray denotes the other tissues.

(1) MRBrainS13 is from the official website [51]. In the training dataset, it has five brain MR images, including 2 male subjects and 3 female subjects, and each subject is associated with 3 modality-channels (i.e., T1, T1_IR, FLAIR) and the manually marked labels of 4 classes, namely, gray matter (GM), white matter (WM), cerebrospinal fluid (CSF), and background, as shown in Figure 6. In the test dataset, it has 30 brain MR images. All the modality has been bias-corrected and the data of each subject is aligned. The voxel size is 0.958 mm×0.958 mm×3 mm for all modalities. Each modality of the MRI data is represented by a 240×240×48 volume;

(2) IBSR18 is also used to evaluate our MSCD-UNet [52]. The IBSR18 training dataset contains 18 subjects, each subject in training data has a single T1-weighted modality. All volumes have a size of 256×256×128 voxels, with voxel space ranging from 0.8 mm×0.8 mm×1.5 mm to 1.0 mm×1.0 mm×1.0 mm. A total of 4 anatomical brain structures are targeted for segmentation.

(3) ISeg2017 is also used to evaluate our MSCD-UNet [53]. ISeg2017 dataset has the combined modalities of T1w and T2w. MRT1 images are obtained with 144 sagittal slices utilizing the following parameters: flip angle = 7°, *TR/TE* = 1900/4.38 ms, and resolution = 1 × 1 × 1 mm^3^. Likewise, MR-T2 images are obtained with 64 axial slices by using: flip angle = 150°, *TR/TE* = 7380/119 ms, and resolution = 1.25 × 1.25 × 1.95 mm^3^. Ten infant subjects with manual labels were provided for training.

### 3.6. Evaluation Metrics

The following common segmentation indicators are employed to evaluate and compare our model with other state-of-the-art methods. The Dice Coefficient (DC), the 95th percentile of the Hausdorff Distance (HD), and the Absolute Volume Difference (AVD) are applied on MRBrainS13 to complete our experiments. For the IBSR18, DC is used for evaluation [54]. For the ISeg2017, DC and ASD is used for evaluation.

Dice coefficient (DC) is defined by the area overlap between the ground truth and segmentation prediction results as:

(8) $$DC\left(G,P\right)=2\frac{G\cap P}{G+P}\times 100\%,$$

where G is the ground truth and P represents the predicted segmentation result. DC is a metric of area overlap between the predicted segmentation result P and the ground truth G.

Because the conventional Hausdorff distance is very sensitive to the outliers, the *K_th_* ranked distance, i.e., $h_{95}=K_{p\in P}^{th}min_{g\in G}∥g-p∥$, is used as to suppress the outliers [52]; it is defined as:

(9) $$HD\left(G,P\right)=max\left\{h_{95}\left(G,P\right),h_{95}\left(P,G\right)\right\},$$

A smaller value $HD\left(G,P\right)$ represents a higher proximity between ground truth and segmentation result.

The absolute volume difference (AVD) is used to evaluate the difference between the predicted volume and the true volume as:

(10) $$AVD\left(G,P\right)=\frac{\left|V_{g}-V_{p}\right|}{Vg}\times 100\%,$$

where $V_{p}$ is the volume of prediction and $V_{g}$ is the volume of truth. A lower value of AVD means the ground truth and prediction result are closer to each other.

The Average Surface Distance (ASD) is used to calculate for the predicted result P and the corresponding ground truth G; it is defined as:

(11) $$ASD\left(G,P\right)=\frac{1}{2}\left(\frac{\sum_{a\in G}min_{b\in p}d\left(a,b\right)}{\sum G}+\frac{\sum_{b\in p}min_{a\in g}d\left(b,a\right)}{\sum P}\right),$$

where $d\left(a,b\right)=∥a-b∥$ represents Euclidean distance between points *a* and *b*.

### 3.7. Implementation Details

Tensorflow is used on the workstation with a NVIDIA GTX_1080Ti GPU in our experiments. In the pre-processing step for the MRBrainS13, IBSR18, and ISeg2017 datasets, MR images are normalized with the zero-mean method, which is calculated as follows: (1) each image is processed by subtracting a Gaussian smoothed image and applying a contrast-limited adaptive histogram equalization to enhance local contrast, (2) the resulting intensity value is subtracted by the mean intensity value and then divided by the standard deviation.

In the training phase, to avoid overfitting, data augmentation techniques (flipping, rotation, elastic stretching, shifting, zoom) are applied in the training procedure to get good performance. The network is trained for 18,000 iterations with ADAM optimizer and Xavier initialization, and the epoch is set as 1. The learning rate is set as 0.001, then being reduced by a factor after every 5000 iterations. Due to the limited capacity of GPU memory, for the input samples and the label samples, both of them with size 32 × 32 × 32, are randomly cropped with a same center point from 4 modalities (T1, FLARI, T1_IR, the label image); thus, they have the corresponding position information. A total of around 72,000 sub-volume samples are extracted by random sampling to feed into the network. For the loss function, the weight of hth output loss function $\beta_{h}$ is set as [1,1,1], the associated weight of the cth class label $\omega_{c}$ is set as [1,1,2,2], and the fusion weight $f_{n}$ is set as [1,1,1].

In the test phase, the final prediction result is obtained by the majority voting strategy on the results of overlapping with a stride of 8.

## 4. Results

We performed an ablation study to investigate the efficacy of employing multi-branch pooling (MP), multi-branch dense prediction (MDP), and multi-branch output module by using five-fold cross-validation.

### 4.1. Ablation for Multi-Branch Pooling (MP)

In order to gain the optimal combination kernel sizes of MP, we enumerated different kernel sizes and test the performance on the MRBrain13 training dataset. We tried different kernel sizes K ranging from 2 to 7 to exploit the optimal combination in the two pooling stages. We named the combination of kernel in the first pooling stage “FP”, and the combination of kernel in the second pooling stage “SP”. In the case K = 7, which roughly equals to the feature map size (8 × 8), the structure becomes “really global pooling”. The results are presented in Table 2. From the results, we can find that the performance is better when the “FP” is the combination kernel size of 5, 3, 2, and “SP” is the combination kernel size of 3, 2. When the “FP” is 2 and “SP” is 2, it represents the standard 3D-UNet.

In additional, in order to exploit the collecting ability of spatial information between max pooling and average pooling, each max pooling was replaced with average pooling in MP. The result of UNet_MP_Aver is shown for MP using average pooling in Table 3. It indicates that the UNet_MP_Max achieves higher performance over the UNet_MP_Aver. Comparing with average pooling, max pooling can effectively reduce the collection of redundant information.

### 4.2. Ablation for Multi-Branch Output with Multi-Branch Dense Prediction (MDP)

As described in Section 3.2, we utilized MDP on the feature maps after using the concatenation layer. To analyze the performance of using MDP at each branch output, Table 4 provides the results of each branch output (B1, B2, B3) with MDP in each scale, in which B1-MDP is 1/4 scale of output, B2-MDP stands for 1/2 scale, and B3-MDP represents 1/1 scale. Additionally, B1, B2, and B3 respectively represent the branch output without MDP. According to the results (displayed in Table 4), it can be seen that the performance is improved by increasing the scale of feature maps and the results of Dice score on WM, GM, and CSF satisfy B1-MDP < B2-MDP < B3-MDP, and B1 < B2 < B3. The fusion of multi-branch output is the key prediction result in the proposed network because it controls the network prediction compensation and performance in different scales. When fusing the branch output prediction with B1-MDP + B2-MDP + B3, named as B4, the segmentation performance is obviously improved for the evaluation metrics on GM and CSF compared with those of two other fusions, B5 (B1 + B2 + B3) and B6 (B1-MDP + B2-MDP + B3 MDP).

**Figure 7:**
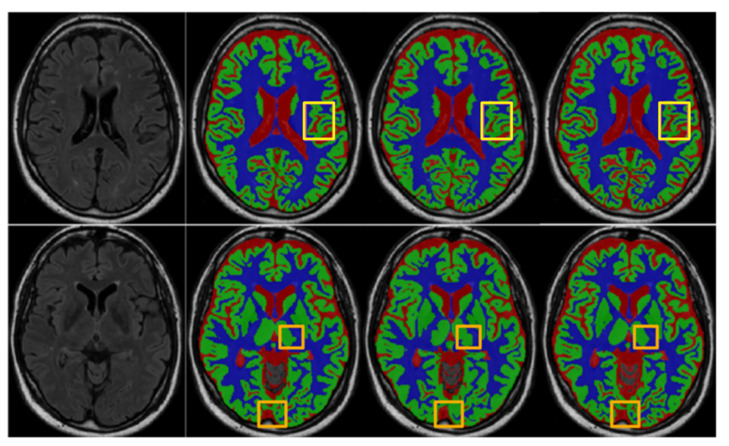
Segmentation results of the UNet and MSCD-UNet on the MR BrainS13 dataset. The rows show the segmentation results of different slices. From first column to last column: FLAIR, manual segmentation, segmentation result of UNet, segmentation result of MSCD-UNet. Center patch in solid yellow box of each segmentation result is highlighted. Each color denotes different brain tissue class, i.e., gray matter (blue), white matter (green), cerebrospinal fluid (red), and other tissues (gray).

Figure 7 provides a visual comparison of the segmentation results produced by the trained UNet and our MSCD-UNet on the MRBrainS13 dataset. It shows that, with MP and MDP, more accurate segmentation results can be generated. Specifically, additional details are preserved, including boundaries and edges.

Finally, it is observed that the result using MSCD-UNet (UNet_MP_Max + B4) is visually more accurate than those of other fusion strategies.

**Figure 8:**
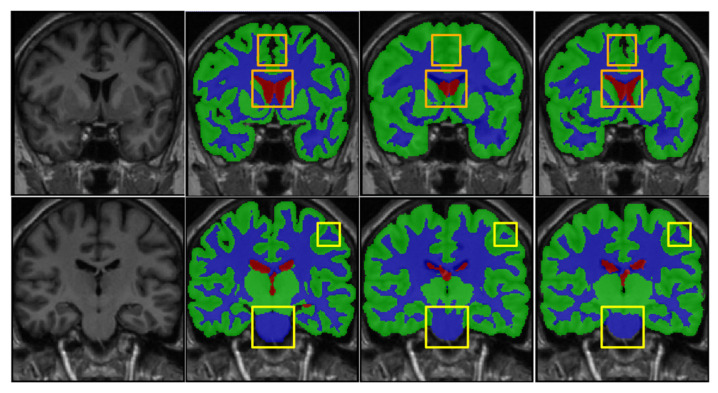
Segmentation results on the IBSR dataset using UNet and MSCD-UNet. The rows show the segmentation results of different slices. From first column to last column: T1, manual segmentation, segmentation result of UNet, segmentation result of MSCD-UNet. Center patch in solid yellow box of each segmentation result is highlighted. Each color denotes different brain tissues classes, i.e., gray matter (green), white matter (blue), cerebrospinal fluid (red), and other tissues (gray).

We also evaluate the MP and MDP on IBSR18 by five-fold cross-validation, where the IBSR18 consists of a larger single-modality T1-weighted MRI with more tissue labels. The evaluation is performed by using five-fold cross-validation on 18 subjects. However, the proposed MSCD-UNet has three channels as the input. Thus, a single subnetwork (e.g., subnetwork for T1 MR images presented in Figure 2) was reserved in MSCD-UNet while the remaining network structures were removed. The results are shown in Table 5. The Dices on GW, WM, and CSF are 85.39%, 89.08%, and 88.14% for UNet, respectively, and 89.82%, 91.18%, and 90.57% for MSCD-UNet, respectively. It reveals that, along with the using of MP and MDP, the performance of MSCD-UNet is obviously improved. Figure 8 provides a visual comparison of the segmentation results produced by the trained UNet and MSCD-UNet on the IBSR18 dataset.

We have evaluated our proposed MSCD-UNet on ISeg2017, where the ISeg2017 consists of T1W, T2W, and label image. Like [44], the evaluation is performed by using nine subjects for training and one subject for validation. We evaluated our results on the ninth subject of the dataset. However, the proposed MSCD-UNet has three channels as the input. Thus, a subnetwork (e.g., subnetwork for T1, FLARI MR images presented in Figure 2) was reserved in MSCD-UNet while the remaining network structures were removed. The results are shown in Table 6. The Dices on GW, WM, and CSF are 91.36%, 89.91%, and 94.70% for UNet, respectively, and 92.17%, 90.47%, and 95.60% for MSCD-UNet, respectively. We can see that using the MP and MDP can yield improvements over the baseline of 3D-UNet.

### 4.3. Comparison with Existing State-of-the-Art Methods

We compare the results between our proposed MSCD-UNet and the state-of-the-art approaches on MRBrainS13 online test dataset. The segmentation of WM, GM, and CSF is evaluated by using the three metrics. A comparison listed in Table 7 indicates that the MSCD-UNet achieves better performance than many state-of-the-art methods [39,40,41,46,55,56]. The reason that our MSCD-UNet performs better is that our model can capture multi-scale information in spatial and channel dimensions by using MP and MDP to alleviate the lack of contextual information and the information loss during the encoding and decoding. Comparing with the similar U-Net architectures [42,48], Li et al. [42] have proposed a Dilated-Inception block to extract multi-scale features from brain MRI; however, it is easy to harness the irrelevant redundant information by using a larger dilation rate. In order to avoid harnessing the irrelevant redundant information, the proposed MP can capture multi-scale feature information with a suitable receptive field. From Table 7, we can see that our proposed architecture achieves better performance than [42]. Sun et al. [48] had the leading method in 2018; however, our proposed method obtained the best score on the GM and CSF, although [48] has a higher score on the CSF. Additionally, our architecture is parameter more efficient compared to [48], with 15 million learned parameters, less than [48], which has 20 million learned parameters. Our proposed multi-branch pooling (MP) and multi-branch dense prediction (MDP) can capture multi-scale feature information with a suitable receptive field, and it is sensitive to segment these brain tissues in edge because the intensity of tissues in edge vary greatly. Thus, our method achieves the best performance on the GM and CSF due to the greatly variation of intensity in the edge.

We also compare the results between our proposed MSCD-UNet and the state-of-the-art approaches on ISeg2017. The segmentation of WM, GM, and CSF is evaluated by using the three metrics. The results are shown in Table 8. The Dices on GW, WM, and CSF are 92.17%, 90.47%, and 95.60%, respectively, for our method. Compared to four other approaches [18,44,45,46], the performance has a higher average Dice score than [45,46]. Although the average Dice is lower than [18], the Dice on GM is higher; additionally, the optimal parameters are waiting to be found, and we will further exploit the potential of MP and MDP in future work.

## 5. Discussion

In this paper, we proposed a Multi-scale Spatial and Channel Dimension-based U-Net for MRI brain segmentation. In our approach, an information extractor multi-branch pooling (MP) is used to capture spatial information in the encoding part, and an information extractor multi-branch dense prediction (MDP) is used to collect as much spatial information as possible into channels in the decoding part. As the intensity of white matter is similar to the gray matter in the rugged edge, enlarging the size of receptive field can improve the recognition performance. In our experiments, we validated that using multiple max pooling with different kernel sizes in parallel can dramatically improve the segmentation performance comparing to the standard 3D U-Net. For example, as shown in Table 2, the Dice coefficients of GM, WM, and CSF by using five-fold cross-validation are 85.94%, 88.83%, and 83.79, respectively, while using the MP can improve the Dice to 86.08%, 89.02%, and 84.15%, respectively. Integration of the multi-scale spatial information in the encoding part can further improve the segmentation accuracy.

Regarding the decoding part, this naive decoding module may not fully recover the segmented object details. During the decoding phase, the compressed feature maps from the deepest encoding layer will be used to recover feature map resolution by using deconvolution and up-sampling. After the maps resolution upsizing, the spatial information in these decompressed feature maps is fixed, so the detailed information is represented more in channel dimension. Hence, it is necessary to collect the complex information in channel dimension. To probe the influence of channel-based multi-scale feature extractor (MDP), we conducted the experiments with and without MDP. The evaluation performance results including DC, HD, and AVD can be seen in Table 4. From these results, we can see the performance of GM, WM, and CSF segmentation improved from 85.94% to 86.41%, 88.83% to 89.18%, and 83.79% to 84.29% on Dice, respectively.

However, our study has some limitations. Although our analysis shows that the MP and MDP with multi-branch output are effective in segmentation of GM, WM, and CSF, if the combination of different kernel sizes in MP and different groups in MDP are selected by a manual setting, which may be tedious and prone to errors if applied in some extreme cases. Nevertheless, this is evidence of the capability of MP and MDP in brain tissue segmentation tasks, indicating the need of further study on this issue to increase the accuracy of such approaches. Another limitation of our model is that it has more than 15 million learned parameters and therefore the training of this model takes more than 8 h. The parameter of the proposed MSCD-UNet is three times larger than the standard 3D-UNet because the MSCD-UNet has three subnetworks for the T1, FLAIR, and T2 in parallel. We used T1, T2, and FLAIR as multi-channel input in the MSCD-UNet, and while the training time was substantially reduced, the performance of segmentation was not satisfactory. Therefore, we should focus on the relationship between this parallel architecture and the performance of segmentation. We believe that the performance of segmentation would be improved, even without this parallel architecture.

## 6. Conclusions

We propose a novel Multi-scale Spatial and Channel Dimension-based U-Net, referred to as MSCD-UNet, by integrating the multi-scale context information in spatial and channel dimensions for brain tissue segmentation. It contains three modules: MP, MDP, and multi-branch output. The MP is an extractor to capture spatial information during the encoding procedure, which consists of multiple max pooling with different kernel sizes in parallel. Extensive experiments indicate that the proposed information extractor MP can effectively enhance the representative ability by exploiting the multi-scale spatial information. The MDP and multi-branch output is a channel-based multi-scale feature extractor, which can recover the corresponding localization to a higher resolution layer in the decoding path. An ablation study demonstrates the effectiveness of the proposed MDP and multi-branch output. This reflects the importance of capturing multi-scale features in enhancing the learning ability in the encoding and decoding paths. We validated our proposed network on the MRBrainS13, IBSR18, and ISeg2017 datasets for brain tissue segmentation and achieved state-of-the-art results as compared to other existing approaches. The proposed method can promote the research on automated brain tissue segmentation as well as offer a useful and effective tool for assessing and diagnosing neurodegenerative diseases and disorders of human brain. In future work, we will explore the proposed network for other medical image challenges.

## Figures and Tables

**Table 1:** An overview of some related works on brain MRI segmentation problems.

Paper	Technique	Advantage	Limitation
[27,28,29,30,31]	Atlas-based registration	Robustness to weak edges, strong adaptability.	Limited by the fuzzy brain tissue edge, multi-source noise, and inhomogeneous intensity.
[31]	SVM	Preserves information in the training images, and easy to implement.	Response time increase dramatically with dataset size. Slow training, memory intensive, and performance patient-specific learning.
[17]	Discriminative dictionary learning		
[32]	Hidden Markov random field		
[33]	Clustering algorithm		
[35,36,37]	Patch-wise CNN	Fast, easy to implement, and low resource hungry. Capture discriminative features from a large input patch.	Sensitive to the patch size, lack of global information, difficult to converge small dataset.
[18,41,43,44,45,46,47]	FCNN with dense connection	Extract more reasonable and contextual information.	Large training time and storage space. High computational complexity.
[48]	FCNN with richer spatial information	Learn required weight for spatial feature extracting.	

**Table 2:** Performances of the combination kernel sizes in the two pooling stages by 5-fold cross-validation in MRBrain13 training dataset (DC:%, HD:mm, AVD:%). The “FP” represents the first pooling stage, the “SP” represents the second pooling stage, and the “K” represents the combination of kernels.

				GM			WM			CSF		
	K		K	DC	HD	AVD	DC	HD	AVD	DC	HD	AVD
FP	7,5,3,2	SP	7,5,3,2	82.47	2.10	7.99	83.59	3.61	7.71	78.22	3.30	8.61
FP	5,3,2	SP	5,3,2	83.12	1.94	7.79	85.40	2.89	7.52	81.45	2.58	8.59
FP	5,3,2	SP	3,2	86.08	1.71	6.76	89.02	1.76	6.71	84.15	2.24	7.82
FP	5,3,2	SP	2	84.50	1.75	7.01	86.04	2.75	7.17	83.23	2.44	8.02
FP	3,2	SP	3,2	85.98	1.90	7.22	88.90	2.32	6.59	84.63	2.18	8.13
FP	3,2	SP	2	82.25	2.03	8.07	84.34	3.48	7.42	83.01	2.98	8.66
FP	2	SP	2	85.94	1.85	7.09	88.83	2.39	6.82	83.79	2.31	8.30

**Table 3:** Performances of UNet, UNet_MP_Max, and UNet_MP_Aver by 5-fold cross-validation (DC:%, HD:mm, AVD:%).

Tissue	GM			WM			CSF		
Evaluation Metric	DC	HD	AVD	DC	HD	AVD	DC	HD	AVD
UNet	85.94	1.85	7.09	88.83	2.39	6.82	83.79	2.31	8.30
UNet_MP_Max	86.08	1.71	6.76	89.02	1.76	6.71	84.15	2.24	7.82
UNet_MP_Aver	85.08	1.98	8.05	88.27	2.23	7.47	82.71	2.70	8.84

**Table 4:** Performances of B1, B2, B3, B1-MDP, B2-MDP, B3-MDP, B4, B5, B6, and MSCD-UNet by 5-fold cross-validation (DC:%, HD:mm, AVD:%).

Tissue	GM			WM			CSF		
Evaluation Metric	DC	HD	AVD	DC	HD	AVD	DC	HD	AVD
B1	72.35	3.12	8.37	75.97	2.79	9.31	70.59	3.78	11.47
B2	75.42	3.06	7.92	79.49	2.37	8.92	77.51	3.69	10.15
B3 (UNet)	85.94	1.85	7.09	88.83	2.39	6.82	83.79	2.31	8.30
B1-MDP	73.04	2.05	8.14	74.33	2.93	9.56	71.06	3.02	9.75
B2-MDP	76.08	2.19	7.66	76.02	2.53	8.74	77.15	3.24	9.82
B3-MDP	85.88	2.01	8.05	88.87	2.23	7.47	83.81	2.70	8.84
B4	86.12	1.91	6.81	88.30	2.06	7.17	83.98	2.23	8.43
B5	85.96	1.93	7.05	89.03	1.88	7.03	83.62	2.40	8.11
B6	85.97	1.99	6.81	89.30	2.09	7.12	83.86	2.31	8.55
MSCD-UNet	86.41	1.52	5.76	89.18	2.13	7.21	84.29	2.16	7.73

**Table 5:** Cross-validation results of MRI brain segmentation using UNet and MSCD-UNet on IBSR18. (DC:%).

Evaluation Metric	DC		
Tissue	GM	WM	CSF
UNet	85.39	89.08	88.14
MSCD-UNet	88.42	90.31	90.57

**Table 6:** The validation results of MRI brain segmentation using UNet and MSCD-UNet on ISeg2017.

Methoed	GM		WM		CSF		Average
	DSC	ASD	DSC	ASD	DSC	ASD	DSC
UNet	0.9136	0.354	0.8991	0.385	0.9470	0.135	0.9136
Ours	0.9217	0.322	0.9047	0.362	0.956	0.110	0.9274

**Table 7:** A comparison with the state-of-the-art methods on MRBrainS2013 online test Dataset.

Tissue	GM			WM			CSF		
Evaluation Metric	DC	HD	AVD	DC	HD	AVD	DC	HD	AVD
**MSCD-UNet**	86.69	1.23	5.65	89.73	1.75	6.21	85.15	1.66	5.70
Sun [48]	86.58	1.29	5.75	89.87	1.73	5.47	84.81	1.84	6.84
Li [42]	86.40	1.38	5.72	89.70	1.88	6.28	84.86	2.03	6.75
Dolz [41]	86.33	1.34	6.19	89.46	1.78	6.03	83.42	2.26	7.31
Chen [40]	86.15	1.44	6.60	89.46	1.93	6.05	87.25	2.19	7.68
Bui [46]	86.06	1.52	6.60	89.00	2.11	5.54	83.76	2.32	6.77
Geraud [39]	86.03	1.44	6.05	89.29	1.86	5.83	82.44	2.28	9.03
Andermatt [55]	85.40	1.54	6.09	88.98	2.02	7.69	84.13	2.17	7.44
Stollenga [56]	84.89	1.67	6.35	88.53	2.07	5.93	83.47	2.22	8.63

**Table 8:** A comparison between proposed architecture and other 3D-based state-of-art methods in terms of DSC and ASD.

Method	GM		WM		CSF		Average
	DSC	ASD	DSC	ASD	DSC	ASD	DSC
Ours	0.9217	0.322	0.9047	0.362	0.956	0.110	0.9274
Lei [44]	0.926	0.307	0.908	0.353	0.959	0.114	0.931
Yu [45]	0.8851	-	0.8546	-	0.9371	-	0.8922
Qamar [18]	0.9205	-	0.9050	-	0.958	-	0.9278
Taoc [46]	0.9157	-	0.9125	-	0.9469	-	0.9250

## Data Availability

Some publicly available datasets were used in this study. This data can be found here: https://mrbrains13.isi.uu.nl/data/ accessed on 7 September 2020, https://www.nitrc.org/frs/?group_id=48 accessed on 7 September 2020, and https://iseg2017.web.unc.edu/ accessed on 29 May 2021.

## References

[1] Wright R., Kyriakopoulou V., Ledig C., Rutherford M., Hajnal J., Rueckert D., Aljabar P. (2014). Automatic quantification of normal cortical folding patterns from fetal brain MRI. Neuroimage.

[2] Wells W., Grimson W., Kikinis R., Jolesz F. (1996). Adaptive segmentation of MRI data. IEEE Trans. Med. Imaging.

[3] Assefa D., Keller H., Ménard C., Laperriere N., Ferrari R.J., Yeung I. (2010). Robust texture features for response monitoring of glioblastoma multiforme on -weighted and -FLAIR MR images: A preliminary investigation in terms of identi-fication and segmentation. Med. Phys..

[4] Qin C., Guerrero R., Bowles C., Chen L., Dickie D.A., del Valdes-Hernandez M., Wardlaw J., Rueckert D. (2018). A large margin algorithm for automated segmentation of white matter hy-perintensity. Pattern Recognit..

[5] Moeskops P., Benders M.J., Chiţǎ S.M., Kersbergen K.J., Groenendaal F., de Vries L.S., Viergever M.A., Išgum I. (2015). Automatic segmentation of MR brain images of preterm infants using supervised classification. Neuroimage.

[6] Maier O., Menze B.H., der Gablentz J., Häni L., Heinrich M.P., Liebrand M., Winzeck S., Basit A., Bentley P., Chen L. (2017). ISLES 2015—A public evaluation benchmark for ischemic stroke lesion segmentation from multispectral MRI. Med. Image Anal..

[7] Janakasudha G., Jayashree P. (2020). Early Detection of Alzheimer’s Disease Using Multi-feature Fusion and an Ensemble of Classifiers. Advanced Computing and Intelligent Engineering.

[8] Ashburner J., Friston K.J. (2005). Unified segmentation. Neuroimage.

[9] Rajchl M., Baxter J.S.H., McLeod A.J., Yuan J., Qiu W., Peters T.M., Khan A.R. ASeTs: MAP-based Brain Tissue Segmentation using Manifold Learning and Hierarchical Max-Flow regularization. Proceedings of the MICCAI Grand Challenge on MR Brain Image Segmentation (MRBrainS’13).

[10] Duchesne S., Pruessner J.C., Collins D.L. (2002). Appearance-Based Segmentation of Medial Temporal Lobe Structures. Neuroimage.

[11] Ballanger B., Tremblay L., Sgambato-Faure V., Beaudoin-Gobert M., Lavenne F., Le Bars D., Costes N. (2013). A multi-atlas based method for automated anatom-ical Macaca fascicularis brain MRI segmentation and PET kinetic extraction. Neuroimage.

[12] Heckemann R.A., Hajnal J.V., Aljabar P., Rueckert D., Hammers A. (2006). Automatic anatomical brain MRI segmentation combining label propagation and decision fusion. Neuroimage.

[13] Scherrer B.F.F., Garbay C., Dojat M. (2009). Distributed local MRF models for tissue and structure brain segmentation. IEEE Trans. Med. Imaging.

[14] Long J., Ma G., Liu H., Song E., Hung C.-C., Xu X., Jin R., Zhuang Y., Liu D. (2020). Cascaded hybrid residual U-Net for glioma segmentation. Multimed. Tools Appl..

[15] Chen H., Qin Z., Ding Y., Tian L., Qin Z. (2020). Brain tumor segmentation with deep convolutional symmetric neural network. Neurocomputing.

[16] Havaei M., Davy A., Warde-Farley D., Biard A., Courville A., Bengio Y., Pal C., Jodoin P.-M., Larochelle H. (2017). Brain tumor segmentation with Deep Neural Networks. Med. Image Anal..

[17] Coupé P., Mansencal B., Clément M., Giraud R., de Senneville B.D., Ta V., Lepetit V., Manjon J.V. (2020). AssemblyNet: A large ensemble of CNNs for 3D whole brain MRI segmentation. Neuroimage.

[18] Qamar S., Jin H., Zheng R., Ahmad P., Usama M. (2020). A variant form of 3D-UNet for infant brain segmenta-tion. Future Gener. Comput. Syst..

[19] Ronneberger O., Fischer P., Brox T. (2015). U-Net: Convolutional Networks for Biomedical Image Segmentation. International Conference on Medical Image Computing and Computer-Assisted Intervention.

[20] Zhao H., Shi J., Qi X., Wang X., Jia J. Pyramid Scene Parsing Network. Proceedings of the 2017 IEEE Conference on Computer Vision and Pattern Recognition (CVPR).

[21] Chen L., Papandreou G., Schroff F., Adam H. (2017). Rethinking Atrous Convolution for Semantic Image Segmentation. arXiv.

[22] Chen L., Zhu Y., Papandreou G., Schroff F., Adam H. Encoder-Decoder with Atrous Separable Convolution for Semantic Image Segmentation. Proceedings of the European Conference on Computer Vision.

[23] Rajawat A.S., Jain S. Fusion Deep Learning Based on Back Propagation Neural Network for Personali-zation. Proceedings of the 2nd International Conference on Data, Engineering and Applications.

[24] Zhang Z., Zhang X., Peng C., Xue X., Sun J. ExFuse: Enhancing Feature Fusion for Semantic Segmenta-tion. Proceedings of the European Conference on Computer Vision (ECCV).

[25] Ma W., Gong C., Xu S., Zhang X. (2020). Multi-scale spatial context-based semantic edge detection. Inf. Fusion.

[26] González-Villà S., Oliver A., Valverde S., Wang L., Zwiggelaar R., Lladó X. (2016). A review on brain structures segmentation in magnetic resonance imaging. Artif. Intell. Med..

[27] Makropoulos A., Counsell S.J., Rueckert D. (2018). A review on automatic fetal and neonatal brain MRI seg-mentation. Neuroimage.

[28] Weisenfeld N.I., Warfield S.K. (2009). Automatic segmentation of newborn brain MRI. Neuroimage.

[29] Anbeek P., Išgum I., Van Kooij B.J.M., Mol C.P., Kersbergen K.J., Groenendaal F., Viergever M.A., De Vries L.S., Benders M.J.N.L. (2013). Automatic Segmentation of Eight Tissue Classes in Neonatal Brain MRI. PLoS ONE.

[30] Artaechevarria X., Munoz-Barrutia A., Ortiz-De-Solorzano C. (2009). Combination Strategies in Multi-Atlas Image Segmentation: Application to Brain MR Data. IEEE Trans. Med. Imaging.

[31] Wang H., Suh J.W., Das S.R., Pluta J.B., Craige C., Yushkevich P.A. (2013). Multi-Atlas Segmentation with Joint Label Fusion. IEEE Trans. Pattern Anal. Mach. Intell..

[32] Wang L., Gao Y., Shi F., Li G., Gilmore J.H., Lin W., Shen D. (2015). LINKS: Learning-based multi-source IntegratioN frameworK for Segmen-tation of infant brain images. Neuroimage.

[33] Zhang Y., Brady M., Smith S. (2001). Segmentation of brain MR images through a hidden Markov random field model and the expectation-maximization algorithm. IEEE Trans. Med. Imaging.

[34] Mishro P.K., Agrawal S., Panda R., Abraham A. (2020). A Novel Type-2 Fuzzy C-Means Clustering for Brain MR Image Segmentation. IEEE Trans. Cybern..

[35] Zhang W., Li R., Deng H., Wang L., Lin W., Ji S., Shen D. (2015). Deep convolutional neural networks for multi-modality isointense infant brain image segmentation. Neuroimage.

[36] Moeskops P., Viergever M.A., Mendrik A.M., De Vries L.S., Benders M.J.N.L., Isgum I. (2016). Automatic Segmentation of MR Brain Images With a Convolutional Neural Network. IEEE Trans. Med. Imaging.

[37] Bao S., Chung A.C. (2018). Multi-scale structured CNN with label consistency for brain MR image seg-mentation. Comput. Methods Biomech. Biomed. Eng. Imaging Vis..

[38] Nie D., Wang L., Gao Y., Shen D. Fully convolutional networks for multi-modality isointense infant brain image segmentation. Proceedings of the 2016 IEEE 13th International Symposium on Biomedical Imaging (ISBI).

[39] Xu Y., Géraud T., Bloch I. From neonatal to adult brain MR image segmentation in a few seconds using 3D-like fully convolutional network and transfer learning. Proceedings of the 2017 IEEE International Conference on Image Processing (ICIP).

[40] Chen H., Dou Q., Yu L., Qin J., Heng P.A. (2017). VoxResNet: Deep voxelwise residual networks for brain seg-mentation from 3D MR images. Neuroimage.

[41] Dolz J., Gopinath K., Yuan J., Lombaert H., Desrosiers C., Ayed I.B. (2019). HyperDense-Net: A Hyper-Densely Connected CNN for Multi-Modal Image Segmentation. IEEE Trans. Med. Imaging.

[42] Li J., Yu Z.L., Gu Z., Liu H., Li Y. (2019). MMAN: Multi-modality aggregation network for brain segmentation from MR images. Neurocomputing.

[43] Chen L., Bentley P., Mori K., Misawa K., Fujiwara M., Rueckert D. (2018). DRINet for Medical Image Segmentation. IEEE Trans. Med. Imaging.

[44] Lei Z., Qi L., Wei Y., Zhou Y., Zhang Y. (2019). Infant Brain MRI Segmentation with Dilated Convolution Pyra-Mid Downsampling and Self-Attention. arXiv.

[45] Yu L., Cheng J.-Z., Dou Q., Yang X., Chen H., Qin J., Heng P.-A. (2017). Automatic 3D Cardiovascular MR Segmentation with Densely-Connected Volumetric ConvNets. Proceedings of the International Conference on Medical Image Computing and Computer-Assisted Intervention.

[46] Bui T.D., Shin J., Moon T. (2017). 3D Densely Convolution Networks for Volumetric Segmentation. arXiv.

[47] Dolz J., Desrosiers C., Wang L., Yuan J., Shen D., Ben Ayed I. (2020). Deep CNN ensembles and suggestive annotations for infant brain MRI segmentation. Comput. Med. Imaging Graph..

[48] Sun L., Ma W., Ding X., Huang Y., Liang D., Paisley J. (2019). A 3D Spatially Weighted Network for Segmentation of Brain Tissue from MRI. IEEE Trans. Med. Imaging.

[49] Maas A.L., Hannun A.Y., Ng A.Y. Rectifier nonlinearities improve neural network acoustic models. Proceedings of the 30th International Conference on Ma-Chine Learning.

[50] Çiçek Ö., Abdulkadir A., Lienkamp S.S., Brox T., Ronneberger O., Ourselin S., Joskowicz L., Sabuncu M., Unal G., Wells W. (2016). 3D U-Net: Learning Dense Volumetric Segmentation from Sparse Annotation. Proceedings of the Medical Image Computing and Computer-Assisted Intervention—MICCAI 2016.

[51] Mendrik A.M., Vincken K.L., Kuijf H.J., Breeuwer M., Bouvy W.H., de Bresser J., Alansary A., de Bruijne M., Carass A., El-Baz A. (2015). MRBrainS challenge: Online Evaluation Framework for Brain Image Segmentation in 3T MRI Scans. Comput. Intell. Neuroence.

[52] Rohlfing T., Brandt R., Menzel R., Maurer C.R. (2004). Evaluation of atlas selection strategies for atlas-based image segmentation with application to confocal microscopy images of bee brains. Neuroimage.

[53] Wang L., Nie D., Li G., Puybareau É., Dolz J., Zhang Q., Wang F., Xia J., Wu Z., Chen J. (2019). Benchmark on Automatic Six-Month-Old Infant Brain Segmentation Algo-rithms: The iSeg-2017 Challenge. IEEE Trans. Med. Imaging.

[54] Rohlfing T. (2012). Image Similarity and Tissue Overlaps as Surrogates for Image Registration Accuracy: Widely Used but Unreliable. IEEE Trans. Med. Imaging.

[55] Andermatt S., Pezold S., Cattin P. (2016). Multi-Dimensional Gated Recurrent Units for the Segmentation of Biomedical 3D-Data. Deep Learning and Data Labeling for Medical Applications.

[56] Stollenga M.F., Byeon W., Liwicki M., Schmidhuber J. (2015). Parallel Multi-Dimensional LSTM, with Appli-Cation to Fast Biomedical Volumetric Image Segmentation. arXiv.

